# A SCOPING REVIEW: EXPLORING COLLABORATIVE GOAL SETTING AMONG AGING ADULTS AND CARE PARTNERS

**DOI:** 10.1093/geroni/igad104.2910

**Published:** 2023-12-21

**Authors:** Kate Perepezko, Tucker Alchin, Bianca Karibian, Catherine Still, Kayla Valente, Pamela Toto, Beth Fields

**Affiliations:** University of Pittsburgh, Pittsburgh, Pennsylvania, United States; University of Pittsburgh, Pittsburgh, Pennsylvania, United States; University of Wisconsin-Madison, Madison, Wisconsin, United States; University of Wisconsin-Madison, Madison, Wisconsin, United States; University of Pittsburgh, Pittsburgh, Pennsylvania, United States; University of Pittsburgh, Pittsburgh, Pennsylvania, United States; University of Wisconsin-Madison, Madison, Wisconsin, United States

## Abstract

Evidence suggests that goal setting and care partner support help aging adults improve their health. Less is known about how aging adults and care partners collaboratively participate in goal setting. The purpose of the current review was to describe the scope of the literature on this topic. A search was conducted in PubMed, CINAHL, SocINDEX, and PscyINFO. Reviewers screened 1,231 articles for the following inclusion criteria: 1) Participants included aging adults (50+ years) and care partners, 2) Goal setting was conducted, and 3) Articles were in English. Of the twenty-nine articles that were included, most (n=21) assessed goal setting for care partners and aging adults separately. Goal setting was primarily directed toward patient care, with only a few studies prompting care partner specific goals (n=4). Common goals reported by aging adults were independence, improving or maintaining functioning, addressing symptoms, and remaining socially active. Care partners listed similar goals but also identified accessing services and supports as important. This review is the first to describe goal setting with aging adults and their care partners, revealing concordant and discordant prioritization of goals within dyads. These findings illustrate the importance and potential complexity of including care partners in the goal setting process. We also found that collaborative goal setting and care partner directed goals are scarce, indicating the need for additional work in this area. Collaborative goal setting aligns with patient and family-centered care approaches and can contribute to better care plans that meet the needs of aging adults and their care partners.

